# Reassessing the temporal evolution of orchids with new fossils and a Bayesian relaxed clock, with implications for the diversification of the rare South American genus *Hoffmannseggella *(Orchidaceae: Epidendroideae)

**DOI:** 10.1186/1471-2148-10-177

**Published:** 2010-06-14

**Authors:** A Lovisa S Gustafsson, Christiano F Verola, Alexandre Antonelli

**Affiliations:** 1 Department of Plant and Environmental Sciences, University of Gothenburg, Box 461, SE-405 30, Göteborg, Sweden; 2 Current Address: National Centre for Biosystematics, Natural History Museum, University of Oslo, P.O. Box 1172 Blindern, 0318 Oslo, Norway; 3 Departamento de Botânica e Ecologia, Instituto de Biociências, Universidade Federal de Mato Grosso, Cuiabá, Av. Fernando Corrêa da Costa s/n, Cuiabá, CEP 78060-900, Mato Grosso, Brazil; 4 Gothenburg Botanical Garden, Carl Skottsbergs gata 22A, 413 19 Göteborg, Sweden

## Abstract

**Background:**

The temporal origin and diversification of orchids (family Orchidaceae) has been subject to intense debate in the last decade. The description of the first reliable fossil in 2007 enabled a direct calibration of the orchid phylogeny, but little attention has been paid to the potential influence of dating methodology in obtaining reliable age estimates. Moreover, two new orchid fossils described in 2009 have not yet been incorporated in a molecular dating analysis. Here we compare the ages of major orchid clades estimated under two widely used methods, a Bayesian relaxed clock implemented in BEAST and Penalized Likelihood implemented in r8s. We then perform a new family-level analysis by integrating all 3 available fossils and using BEAST. To evaluate how the newly estimated ages may influence the evolutionary interpretation of a species-level phylogeny, we assess divergence times for the South American genus *Hoffmannseggella *(subfam. Epidendroideae), for which we present an almost complete phylogeny (40 out of 41 species sampled).

**Results:**

Our results provide additional support that all extant orchids shared a most recent common ancestor in the Late Cretaceous (~77 million years ago, Ma). However, we estimate the crown age of the five orchid subfamilies to be generally (~1-8 Ma) younger than previously calculated under the Penalized Likelihood algorithm and using a single internal fossil calibration. The crown age of *Hoffmannseggella *is estimated here at ~11 Ma, some 3 Ma more recently than estimated under Penalized Likelihood.

**Conclusions:**

Contrary to recent suggestions that orchid diversification began in a period of global warming, our results place the onset of diversification of the largest orchid subfamilies (Orchidoideae and Epidendroideae) in a period of global cooling subsequent to the Early Eocene Climatic Optimum. The diversification of *Hoffmannseggella *appears even more correlated to late Tertiary climatic fluctuations than previously suggested. With the incorporation of new fossils in the orchid phylogeny and the use of a method that is arguably more adequate given the present data, our results represent the most up-to-date estimate of divergence times in orchids.

## Background

Orchidaceae is the largest and one of the ecologically and morphologically most diverse families of flowering plants [1]. Several ages have been proposed for the origin of modern orchid lineages (i.e., their crown age), ranging from ~26 million years (Ma) [2], ~40 Ma [3], ~80 Ma [4] to as much as ~110 Ma [5]. A correct time estimation is essential for our understanding of the mechanisms underlying the diversification of orchids, and could contribute to discern between alternative hypotheses of diversification - such as significant increases in speciation rates temporally correlated to climatic changes, tectonic events, or radiation of pollinators.

Many parameters have been identified to affect divergence time estimates in phylogenies, including taxon sampling, reliability, number and placement of internal calibration points, and dating method [6-16]. Until recently, molecular dating of the Orchidaceae has been challenging due the complete absence of reliable orchid fossils. The finding of a 15-20 Ma fossil of an extinct stingless bee (*Proplebeia dominicana*), covered with pollinia from an orchid species belonging to the subtribe Goodyerinae, finally allowed for temporal calibration of the family [4]. Using this fossil as an internal calibration point, and departing from a phylogenetic tree obtained from the analysis of plastid DNA sequences (*mat*K and *rbc*L), Ramirez *et al. *[4] estimated the origin of Orchidaceae at 76-84 Ma. These results supported an 'ancient' origin of orchids in the Late Cretaceous.

Although the study by Ramirez *et al. *[4] unquestionably constituted a milestone in orchid research, the large discrepancies in age estimates obtained in the last decade - some 80 Ma between the youngest [2] and oldest [5] crown ages - suggests that the matter is probably not completely settled. In a recent study in the family Begoniaceae, Goodall-Copestake *et al.*[16] found that the second largest source of variance in age estimates (after availability and placement of internal calibration points) was derived from the choice of dating method employed, a result consistent with previous evaluations of empirical data [10,11]. In particular, recent developments in molecular dating techniques have called into question the assumptions and algorithms implemented in Non-parametric Rate Smoothing [NPRS; [17]] and Penalized Likelihood [PL; [18]] - the two methods employed by Ramirez *et al. *[4]. Whereas NPRS has been largely abandoned in favour of its successor PL [see discussion in [18]], both implemented in the software r8s [19], PL competes today in popularity with Bayesian dating [20] implemented in the software BEAST [21].

PL and BEAST operate in very different ways: *i) *PL requires a fixed phylogram as input, whereas BEAST samples topologies simultaneously as it calculates divergence times under a MCMC analysis, and allows the choice of several different priors and models; *ii) *PL assumes autocorrelation of rates within the phylogeny (i.e. that mutational rates are inherited, resulting in closely related taxa exhibiting similar evolutionary rates), whereas BEAST allows branches to vary in evolutionary rate; *iii) *in PL, nodes can be calibrated to be either fixed to a certain age, or constrained by a maximum or a minimal bound; whereas in BEAST, several additional alternatives are available for calibrating a node, because such calibrations represent age priors drawn from distributions of various shapes (e.g., normal, lognormal, exponential, or uniform). The methodological and conceptual differences between r8s, BEAST, and some other methods available today for molecular dating have been reviewed by several authors [12,14,22-24].

Although the methodology and assumptions implemented in each molecular dating method can be readily compared, our knowledge of how time estimates are influenced by the choice of method is still poor. For instance, Goodall-Copestake *et al.*[16] obtained younger ages in the Begoniaceae using PL than using NPRS, but as these authors noted the inverse situation was found by Clement *et al. *[25,26] on the same taxonomic group. According to Goodall-Copestake [16], this surprising discrepancy was probably caused by differences in density of sampled taxa and calibration points. Similarly, it may be very difficult to predict differences in age estimates using PL and BEAST: in the study by Goodall-Copestake [16], PL produced considerably younger ages than BEAST, whilst the opposite situation was found within family Caryophyllaceae [26]. These results exemplify the potential influence of methodology on age estimations.

In this study we aim at reassessing the temporal origin and diversification of Orchidaceae, using the Bayesian uncorrelated relaxed molecular clock approach implemented in BEAST. In addition to choosing a different dating method, we conduct a new analysis on an expanded taxon sampling by adding two internal calibration points in the orchid phylogeny. We base these calibrations on fossil leaves described subsequent to the study by Ramirez *et al. *[4] from Early Miocene deposits of New Zealand, which were confidently assigned to genera *Dendrobium *and *Earina *[27]. Then, to explore how the high-level age estimates obtained here may affect the evolutionary interpretation of a species-level orchid clade, we date the origin and diversification of the rare South American orchid genus *Hoffmannseggella*.

*Hoffmannseggella *belongs to the Epidendroideae, the largest subfamily within Orchidaceae, which comprises over half of all orchid species [28]. The subfamily has been divided into 'lower' and 'higher' Epidendroids [29] and this latter clade includes the monophyletic subtribe Laeliinae, where *Hoffmannseggella *is nested [30]. The genus is endemic to Brazil, where it is confined to the High Altitude Rocky Complexes (Brazilian Campos Rupestres and Campos de Altitude) of Minas Gerais, Rio de Janeiro, Espírito Santos and Bahia states. It comprises exclusively rupicolous species, i.e. growing among rocks [31]. Adding to the 32 different species recognized by Chiron and Castro Neto [32], several new species have recently been described and today *Hoffmannseggella *comprises 41 species [31,33-39]. Half of these are "micro-endemic" - known from a single natural population, and some only from the type collection. We have been able to obtain or generate sequences for all but a single species, thus reaching a 98% complete species sampling.

## Results

**Figure 1:**
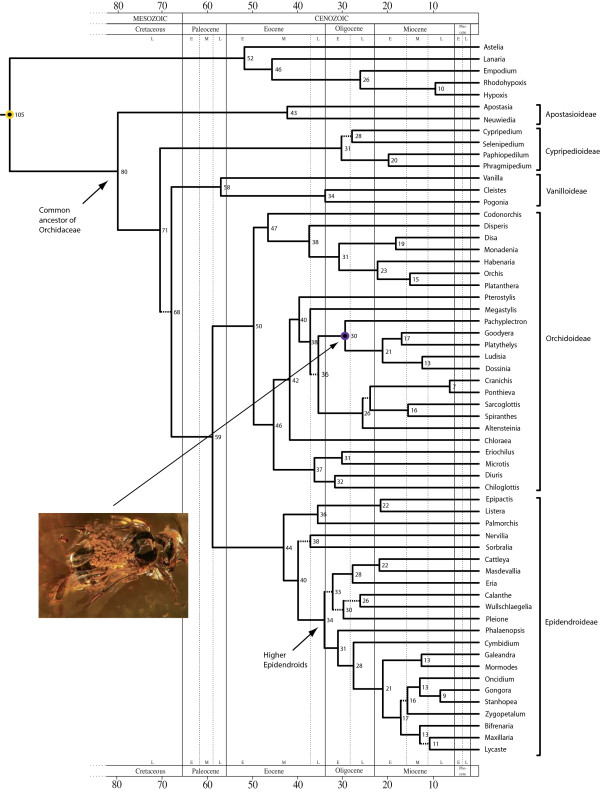
**Time-calibrated tree of the Orchidaceae based on plastid DNA sequences (*mat*K and *rbc*L), obtained with BEAST, using the same matrix and calibration points as Ramirez *et al***. [4]. Numbers at nodes are median ages in million of years (Ma). Dashed branches indicate nodes with Bayesian posterior probabilities below 0.90. Circles indicate age-constrained nodes; the yellow circle indicates the root node, the purple circle indicates the internal calibration point for subtribe Goodyerinae (*Pachyplectron - Dossinia*). **Inset: **The extinct stingless bee *Proplebeia dominicana *with well-preserved pollinia attached to the mesoscutellum; these pollinia have been demonstrated to belong to an extinct orchid species in subtribe Goodyerinae. This represents the first definitive fossil record for the Orchidaceae. Reprinted by permission from Macmillan Publishers Ltd: Nature 448(30), copyright 2007.

The maximum credibility tree for the fossil-calibrated relaxed molecular clock analysis of the family Orchidaceae, using the same matrix and calibration points as Ramirez *et al. *[4], is shown in Figure 1. Support values for the different clades and branches were high, with only a few values below 0.90 Bayesian posterior probability. Our age estimates indicate that extant Orchidaceae shared a most recent common ancestor (MRCA) in the Late Cretaceous, ~80 Ma (95% confidence intervals, CI: 56 - 105 Ma). Accordingly, median divergence times for the five orchid subfamilies currently recognized (Apostasioideae, Vanilloideae, Cypripedioideae, Orchidoideae and Epidendroideae) varied between ~31 Ma and ~58 Ma (Table 1). The most diverse orchid subfamilies Orchidoideae and Epidendroideae appear to have started to diversify in the Eocene, 50 Ma (95% CI: 34 - 67 Ma) and 44 Ma (95% CI: 29 - 60 Ma), respectively (Table 1).

**Table 1:** Crown group ages (in million of years) estimated for the family Orchidaceae, the five orchid subfamilies, the 'Higher Epidendroids' and the subtribe Goodyerinae, as compared to previous estimates using Penalized Likelihood [4].

Clade	**Ramirez *et al. ***[4]**: Penalized Likelihood**	**This study: BEAST, same data set as Ramirez *et al. ***[4]	This study: BEAST, with additional taxa and calibration points
	
	Oldest & youngest mean ages (± standard deviations)	Median (95% HPD)	Median (95% HPD)
Family **Orchidaceae**	84 ± 6; 76 ± 5	80 (56-105)	77 (63-92)
Subfamily **Apostasioideae**	49 ± 5; 45 ± 4	43 (23-66)	41 (23-61)
Subfamily **Vanilloideae**	71 ± 5; 65 ± 4	58 (39-79)	57 (43-72)
Subfamily **Cypripedioideae**	37 ± 4; 34 ± 4	31 (17-49)	33 (19-50)
Subfamily **Orchidoideae**	58 ± 5; 52 ± 4	50 (34-67)	53 (42-64)
Subfamily **Epidendroideae**	59 ± 8; 51 ± 7	44 (29-60)	49 (38-62)
**'Higher Epidendroids'**	50 ± 7; 42 ± 6	34 (22-45)	39 (31-49)
Subtribe **Goodyerinae**	38 ± 4; 34 ± 3	30 (20-42)	32 (23-41)
*Dendrobium*/*Earina*	n/a	n/a	32 (25-40)

**Figure 2:**
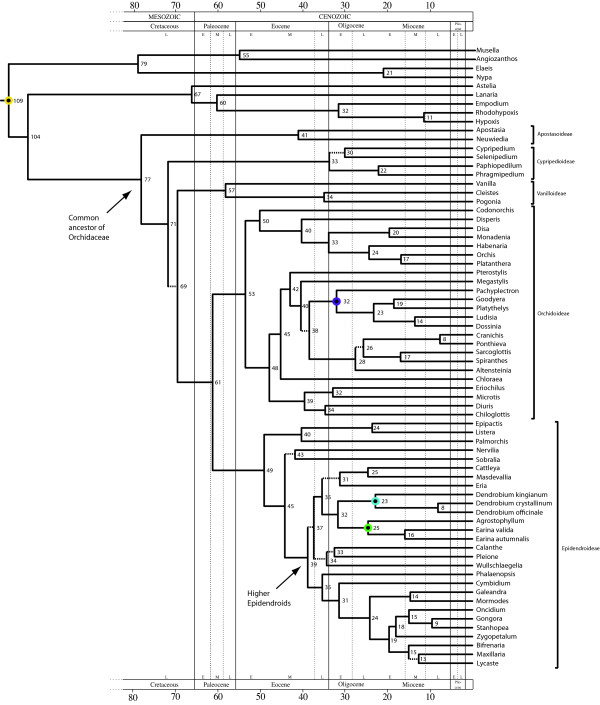
**Time-calibrated tree of the Orchidaceae based on plastid DNA sequences (*mat*K and *rbc*L) obtained with BEAST, including two new calibration points (*Dendrobium *and *Earina*), and with the root calibration on the stem node of Asparagales**. Numbers at nodes are median ages in million of years (Ma). Dashed branches indicate nodes with Bayesian posterior probabilities below 0.90. Circles indicate age-constrained nodes; the yellow circle indicates the root node, the purple circle indicates the internal calibration point for subtribe Goodyerinae (*Pachyplectron - Dossinia*), the blue circle indicates the new internal calibration point for *Dendrobium *and the green circle indicates the new internal calibration point for *Earina*.

Figure 2 shows the maximum credibility tree for the fossil-calibrated relaxed molecular clock analysis of the family Orchidaceae including the two new calibration points (*Dendrobium *and *Earina*). Also in this analysis the support values for most clades were high, with only a few values below 0.90 Bayesian posterior probability. In agreement with the previous analysis, our age estimates indicate that extant Orchidaceae shared a MRCA in the Late Cretaceous, ~77 Ma (95% confidence intervals, CI: 63 - 92 Ma). Median divergence times for the five orchid subfamilies ranged from 33 Ma to 57 Ma (Table 1). The most diverse orchid subfamilies Orchidoideae and Epidendroideae are inferred also in this analysis to have started to diversify in the Eocene, 53 Ma (95% CI: 42 - 64 Ma) and 49 Ma (95% CI: 38 - 62 Ma), respectively (Table 1). The median age for the crown 'Higher Epidendroids' was 39 Ma, with a 95% CI of 31 - 49 Ma. The median divergence time for the node separating *Dendrobium */*Earina *was 32 Ma, with a 95% CI of 25 - 40 Ma (Table 1).

**Figure 3:**
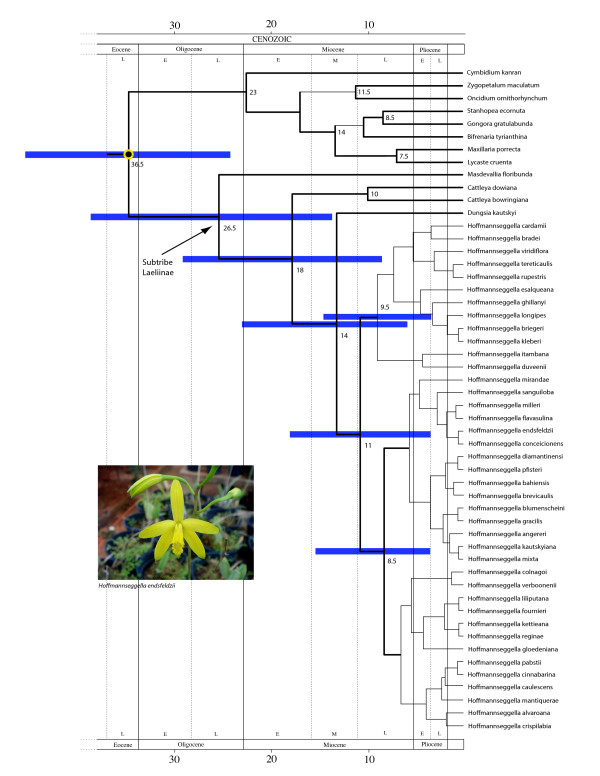
**Time-calibrated tree of the 'Higher Epidendroids', focusing on the South American orchid genus *Hoffmannseggella*, based on nuclear ribosomal DNA sequences (nrITS), estimated with BEAST**. Thin branches indicate posterior probabilities below 0.9. Numbers at nodes are median ages in million of years (Ma). Node bars indicate the 95% HPD lower and upper bounds in Ma. Inset: *Hoffmannseggella endsfeldzii*, showing the racemose inflorescence typical to the genus, and brightly yellow-coloured flowers. Photo: ALSG.

The maximum credibility tree for the calibrated relaxed molecular clock analysis of the 'Higher Epidendroids' including the genus *Hoffmannseggella *is shown in Figure 3. All *Hoffmannseggella *species clustered together with high support value (posterior probability above 0.90), supporting the monophyly of the genus. However, the resolution within the genus was poor, mainly distinguishing two subclades. According to our dating, the genus *Hoffmannseggella *shared a MRCA in the Late Miocene, ~11 Ma (95% CI: 5 - 20 Ma) (Figure 3).

## Discussion

### Evolution of major orchid clades

According to our new estimates based on the uncorrelated relaxed molecular clock approach, and incorporating three internal calibration points, extant Orchidaceae shared a MRCA in the Late Cretaceous, about 77 Ma (95% CI: 63 - 92 Ma). Except for Orchidoideae, median age estimates for the remaining orchid subfamilies are consistently younger in our study as compared to the youngest mean ages obtained by Ramirez *et al. *[4] (Table 1). Also in the first analysis where we used the same matrix as Ramirez *et al. *[4] and obtained precisely the same topology for the phylogenetic tree, median age estimates for the five orchid subfamilies are younger (Table 1).

**Figure 4:**
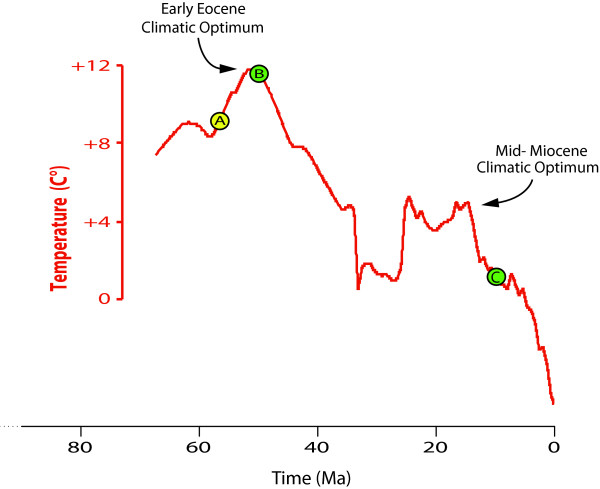
**Temperature curve over the past 65 million years (adapted from Zachos *et al. ***[40]**), illustrating the different results obtained here using BEAST as compared to previous estimates under the Penalized Likelihood algorithm**. (A): Mean age of the major diversification of Orchidaceae (subfamilies Orchidoideae and Epidendroideae) as estimated by Ramirez *et al. *[4] using PL. (B): approximate median age of these clades estimated here using BEAST. (C): median age of the South American genus *Hoffmannseggella *in the Late Miocene, estimated here. The temperature scale is relative to the current mean global temperature ([41] provides an explanation and expanded data, but not a mean temperature curve).

Ramirez *et al. *[4] proposed a Late Palaeocene radiation (~56 Ma) for the Orchidaceae, thus during the prominent temperature increase of the Early Cenozoic (from 59 Ma to 52 Ma; [40,41]). In contrast, our results suggest a younger diversification, placing the origin of the two largest orchid subfamilies Orchidoideae and Epidendroideae in the Eocene (~53 Ma and ~49 Ma, respectively), thus at the onset of a long period of temperature decrease [40,41] (Figure 4). If these estimates are correct, a potential explanation for the initial radiation of Orchidaceae during the Eocene could be that cooler temperatures increased the global heterogeneity of ecosystems (e.g., with more open and dry habitats), creating new habitats that could foster adaptive radiation and/or increasing allopatric speciation (e.g.[42]). An alternative or complementary scenario is that the Early Eocene Climatic Optimum 51-53 Ma [40,41], when mean global temperatures reached ~12°C higher than today's level (Figure 4), would have caused a wave of large-scale extinction and thus left many empty ecological niches available for orchid diversification.

### *Radiation of the genus *Hoffmannseggella

Our estimate for the crown age of *Hoffmannseggella *indicates a Late Miocene radiation for the genus (~11 Ma; Figure 3). This is some 3 Ma younger than the dates obtained using a Penalized Likelihood analysis over a sample of Bayesian phylograms [43,44] (mean 14.2 Ma, 95% CI: 9.69 - 18.6).

Antonelli *et al. *[44] postulated a strong correlation between climate cooling following the Mid-Miocene Climatic Optimum and range expansion and diversification in *Hoffmannseggella*. This younger age estimate may imply an even stronger link than originally conceived, since the intensity of climatic oscillations augmented towards the end of the Tertiary [40,41].

### Reliability of results

The age estimation of genus *Hoffmannseggella *is based on a single, secondary calibration point (the crown group age of 'Higher Epidendroids' obtained in the high-level dating analysis), which should have a direct effect on all internal divergence times. However, the node age estimations between *Cattleya *and *Masdevallia *are strikingly similar in both analyses (25 Ma in the high-level Orchidaceae data set, CI:14 - 35; and 26.5 Ma in the *Hoffmannseggella *data set, CI: 14 - 41). This agreement provides some cross-validation for the use of the 'Higher Epidendroids' as a calibration point for the *Hoffmannseggella *data set.

As outlined in the Introduction, PL and BEAST make different evolutionary assumptions and have very different algorithms. This precludes categorical assertions on which of these methods yields the most correct divergence time estimates. One way to assess the autocorrelation assumption made by PL is to examine the covariance between parent and child branch in each phylogeny. This value is calculated by the software Tracer v1.4 [45] from the log files of the MCMC analyses, and should be significantly positive when rates are autocorrelated, and near zero when there is no evidence of autocorrelation (see BEAST manual). For the Orchidaceae data set, this covariance had a mean of 0.10 and 95% confidence intervals ranging from -0.05 to 0.26. Although the covariance has been criticized as a weak measure of autocorrelation and more critical discussion on this subject is needed [46], the low covariance found in this study does not provide positive evidence for autocorrelation, thus favouring the BEAST results reported here.

### Influence of internal fossil calibrations

**Figure 5:**
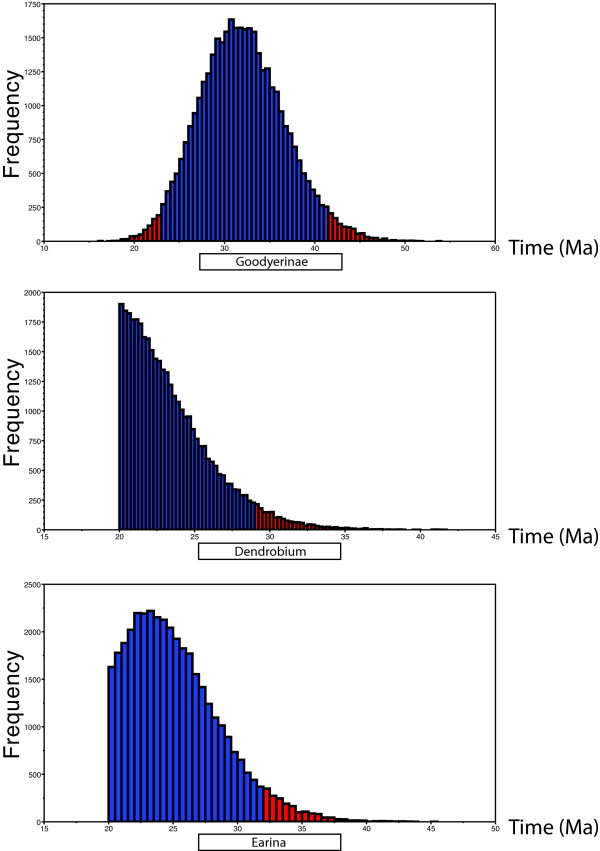
**Posterior age distribution of the three fossil-calibrated internal nodes in the orchid phylogeny (outlined in Figure 2)**. The diagrams show the age of the most recent common ancestor of these nodes (in millions of years) plotted against frequency of trees, from the combined results of 5 independent BEAST runs of 20 million generations each (burn-in excluded). (A) Crown age of tribe Goodyerinae, constrained to a minimal age of 15 Ma. (B) Crown age of genus *Dendrobium*, constrained to a minimal age of 20 Ma. (C) Crown age of genera *Earina *and *Agrostophyllum *(in this analysis, equivalent to stem age of *Earina*), constrained to a minimal age of 20 Ma.

It is worth noting that the single fossil used by Ramirez *et al. *[4], for tribe Goodyerinae, did not influence any age estimates for Orchidaceae in the BEAST analysis. This is evident by examining the distribution of ages of the MRCA of tribe Goodyerinae, which falls outside the lower age prior of 15 Ma (Figure 5a). In contrast, both New Zealand fossils affected age estimates, as is apparent from the age distributions of the two constrained nodes, truncated at their younger bound 20 Ma (Figure 5b, c). While the *Dendrobium *crown age constraint was most frequently reached during the analyses (Figure 5b), the *Earina *stem age constraint was often reached but did not prevent a normally distributed age distribution with a mean older than the fossil constraint (Figure 5c).

## Conclusions

Molecular dating techniques have greatly improved in the last years, offering novel opportunities to study the temporal evolution of taxa. However, it is essential to critically evaluate the impact of methodology and other parameters (taxon sampling, fossil calibrations, sequence regions) on the reliability of results. This study has shown that age estimations for orchid clades vary by several million years when using BEAST or Penalized Likelihood. While the addition of two new internal calibration points makes our study the most up-to-date estimate of the temporal evolution of orchids, additional studies may be required before a stable chronogram of this charismatic plant family is achieved.

## Methods

### Taxon sampling and genetic markers

In the first analysis for the Orchidaceae family, we used the matrix compiled by Ramirez *et al. *[4], including aligned plastid DNA sequences (*mat*K and *rbc*L). The matrix comprised 60 taxa and 2858 characters (see [4] for a detailed list of included species and GenBank accession numbers). In the second dating analysis of the Orchidaceae, we used six additional taxa, including sequences of both *mat*K and *rbc*L, to allow the use of two recently described orchid fossils from New Zealand as calibration points. We also added sequences from four new outgroup genera, which enabled us to use the stem node of Asparagales as root calibration point, instead of the crown node, as done by Ramirez *et al. *[4]. We did this because the oldest fossil used by Ramirez *et al. *[4] was *Liliacidites sp*., which does not have any diagnostic features of crown Asparagales. The additional sequences were re-aligned with those of Ramirez *et al. *[4], using MAFFT version 5.64 [47]. The final matrix comprised 70 taxa and 2905 characters (see Table 2 for the additional species and their corresponding GenBank accession numbers).

**Table 2:** Species list for the additional species included in the new Orchidaceae dataset, with GenBank accession numbers.

*Species*	*rbcL*	*matK*
1. *Agrostophyllum majus *Hook. f.	AF518054.1	AY368391.1
2. *Anigozanthos flavidus *DC.	AJ404843.1	AB088796.1
3. *Dendrobium crystallinum *Rchb. f.	D58407.1	AF445447.1
4. *Dendrobium kingianum *Bidwill ex Lindl.	AF074146.1	AF263651.1
5. *Dendrobium officinale *Kimura & Migo	FJ216567.1	FJ794044.1
6. *Earina autumnalis *Hook. f.	AF074155.1	AF263656.1
7. *Earina valida *Rchb. f.	AF518051.1	AY121741.1
8. *Elaeis oleifera *(Kunth) Cortés	AY012509.1	EU016887.1
9. *Musella lasiocarpa *(Franch.) H.W.Li.	AF243844.1	AF478909.1
10. *Nypa fruticans *Wurmb	M81813.1	AF543743.1

For *Hoffmannseggella*, 40 out of 41 species were sampled (Table 3). The ITS region (ITS1-5.8S-ITS2) was sequenced for 14 species to complement the study by van den Berg *et al. *[30]. The matrix, including outgroup species selected on the basis of previous phylogenetic analyses [4,30], comprised 56 taxa and 660 characters. No gaps were coded in any of the matrices.

**Table 3:** Species list for the *Hoffmannseggella *data set with voucher and GenBank accession numbers, indicating ITS sequences obtained from van den Berg [28] and the species sequenced for this study.

*Species*	*Voucher*	*GenBank No.*	***Included in ***[28].	*New sequences*
1. *H. alvaroana *F. E. L. Miranda	van den Berg C227 (ESA)	AY008672	x	
2. *H. angereri *Pabst	C223-Machado s. n. (ESA)	AY008652	x	
3. *H. bahiensis *Schultr.	C221-Machado s. n. (ESA)	AY008653	x	
4. *H. blumenscheinii *Pabst	C209-Machado s. n. (ESA)	AY008654	x	
5. *H. bradei *Pabst	C215-Machado s. n. (ESA)	AY008673	x	
6. *H. brevicaulis *(H. G. Jones) Withner	C208-Machado s. n. (ESA)	AY008655	x	
7. *H. briegeri *Blumensch. ex Pabst	Brieger Coll. 4612 (ESA)	AY008674	x	
8. *H. cardimii *Pabst & A. F. Mello	C205-Machado s. n. (ESA)	AY008675	x	
9. *H. caulescens *Lindl.	Brieger Coll. 1916 (ESA)	AY008656	x	
10. *H. cinnabarina *Batem. Ex Lindl	Brieger Coll. 1395 (ESA)	AY008657	x	
11. *H. colnagoi *Chiron & astro	A. L. S. Gustafsson 09 (GB)	FJ200183		x
12. *H. conceicionensis *Castro & ampacci	A. L. S. Gustafsson 14 (GB)	FJ200196		x
13. *H. crispilabia *(A. Rich. ex Rchb. f.) Warner	Brieger Coll. 4837 (ESA)	AY008659	x	
14. *H. diamantinensis *Castro & Marçal	A. L. S. Gustafsson 08 (GB)	FJ200187		x
15. *H. duveenii *Fowlie	C213-Machado s. n. (ESA)	AY008671	x	
16. *H. endsfeldzii *(Pabst) Castro & hiron	A. L. S. Gustafsson 06 (GB)	FJ200190		x
17. *H. esalqueana *Blumensch. Ex Pabst	Brieger Coll. 4980 (ESA)	AF260198	x	
18. *H. flavasulina *Miranda & acerda	A. L. S. Gustafsson 05 (GB)	FJ200186		x
19. *H. fournieri *(Cogniaux) Castro & hiron	A. L. S. Gustafsson 01 (GB)	FJ200185		x
20. *H. ghillanyi *Pabst	C214-Machado s. n. (ESA)	AY008677	x	
21. *H. gloedeniana *Hoehne	van den Berg C35(ESA)	AY008666	x	
22. *H. gracilis *(Pabst) Castro & hiron	A. L. S. Gustafsson 04 (GB)	FJ200191		x
23. *H. itambana *Pabst	C-Machado s. n. (ESA)	AY008678	x	
24. *H. kautskyana *Castro & hiron	A. L. S. Gustafsson 10 (GB)	FJ200188		x
25. *H. kettieana *Pabst	C210-Machado s. n. (ESA)	AY008664	x	
26. *H. kleberi *Miranda	A. L. S. Gustafsson 11 (GB)	FJ200194		x
27. *H. liliputana *Pabst	A. L. S. Gustafsson 03 (GB)	FJ200189		x
28. *H. longipes *Rchb. f.	Brieger Coll. 5183 (ESA)	AY008676	x	
29. *H. mantiqueirae *Pabst ex D. C. Zappi	van den Berg C224 (ESA)	AY008663	x	
30. *H. milleri *Blumensch. ex Pabst	Brieger Coll. 5070 (ESA)	AY008661	x	
31. *H. mirandae *Lacerda & astro	A. L. S. Gustafsson 07 (GB)	FJ200193		x
32. *H. mixta *Hoehne ex Ruschi	C220-Machado s. n. (ESA)	AY008660	x	
33. *H. pabstii *Miranda & acerdo	A. L. S. Gustafsson 12 (GB)	FJ200184		x
34. *H. pfisteri *Pabst & Senghas	van den Berg C226 (ESA)	AY008662	x	
35. *H. presidentensis *Camacci	***not available for sequencing***			
36. *H. reginae *Pabst	C218-Machado s. n. (ESA)	AY008668	x	
37. *H. rupestris *Lindl.	van den Berg C33 (ESA)	data missing	x	
38. *H. sanguiloba *Withner	C216-Machado s. n. (ESA)	AY008669	x	
39. *H. tereticaulis *Hoehne	van den Berg C222 (ESA)	AY008670	x	
40. *H. verboonenii *(Miranda) Castro & hiron	A. L. S. Gustafsson 13 (GB)	FJ200192		X
41. *H. viridiflora *Verola & Semir	A. L. S. Gustafsson 02 (GB)	FJ200195		X
42. *Cattleya dowiana *Batem	Chase O-282 (K)	AF260210	(X) only *rbc*L	
43. *Cattleya bowringiana *Veitch	Brieger Coll. 96 (ESA)	AY008585		
44. *Dungsia kautskyi *Pabst	van den Berg C286 (K spirit)	AY008651		
45. *Masdevallia floribunda *Lindl.	Chase O-296 (K)	AF260146	X	
46. *Cymbidium kanran *Makino	data missing	AF284720		
47. *Oncidium ornithorhynchum *Kunth	data missing	AF239400		
48. *Gongora gratulabunda *Rchb.f.	data missing	AF239382		
49. *Stanhopea ecornuta *Lem.	data missing	AF239349		
50. *Zygopetalum maculatum *(Kunth) Garay	Chase 160 (K)	AY870097		
51. *Bifrenaria tyrianthina *Rchb. f.	data missing	DQ210235		
52. *Maxillaria porrecta *Lindl.	data missing	DQ210568		
53. *Lycaste cruenta *Lindl.	data missing	AF239342		

### DNA extraction, amplification and sequencing

Total genomic DNA was extracted exclusively from fresh plant material using a 2% CTAB protocol (adapted from [48]). Amplification was performed using PuReTaq™Ready-To-Go™PCR beads (Amersham Biosciences) for 25 μL reactions using 20 pmol of each primer. The two primers used were 'P17' (5'-CTACCGATTGAATGGTCCGGTGAA-3') and '26S-82R' (5'-TCCCGGTTCGCTCGCCGTTACTA3') of [49]. PCR-products were analysed by electrophoresis using a 1% agarose gel and purified using QIAquick^® ^PCR Purification Spin Columns (QIAGEN^®^). Quantification of the PCR-products was then done using GeneQuant II (Pharmacia Biotech).

Sequencing was performed using a CEQ™8000 (Genetic Analysis System, software 8.0, Beckham Coulter^®^) automated sequencer. Reactions were made with GenomeLab™DTCS-Quick Start Kit (Beckham Coulter^®^) according to manufacturer's instructions, except that 10 μL reactions were used, with 50 ng template and 1.6 pmol per reaction. The two primers used for sequencing ITS were 'P16b' (5'-CCAYTGAACCTTATCATTKAGAGGA-3') of [50] and 'ITS4R' (5'- TCCTCCGCTTATTGATATGC-3') of [51]. Editing and compilation of the sequences was done using Sequencher™version 4.1 (Gene Codes Corporation).

### Sequence alignment and dating analyses

The matrix for Orchidaceae [4] was analysed using a relaxed molecular clock approach with the software BEAST v1.5.3 [21]. The input data were compiled in BEAUti v1.5.3 with the tree priors set as follows: *i) *age for the monophyletic subtribe Goodyerinae (corresponding to the age of the fossil orchid pollinia 15 - 20 Ma old; [4]): uniform prior distribution with a lower bound of 15 Ma and an upper bound of 120 Ma; *ii) *age for the root of the tree (corresponding to the oldest known fossil record for Asparagales; see discussion in [4]): normal prior distribution with mean 106.5 Ma and standard deviation of 8.21 (giving a 95% CI ranging from 93 - 120 Ma). The second family-level matrix (with the additional taxa) was analysed with the following tree priors: *i) *age for the monophyletic subtribe Goodyerinae set as above; *ii) *the two additional calibration points for *Dendrobium *and *Earina *set as uniform prior distributions with a lower bound of 20 Ma and an upper bound of 120 Ma (phylogenetic placement following [27]); *iii) *age for the root of the tree set to an uniform prior distribution with a lower bound of 93 Ma and an upper bound of 120 Ma. The upper (maximum) age constraint of 120 Ma for the calibrations above corresponds to the oldest known monocot fossils [52]. We acknowledge that this constraint may be questionable since fossils generally provide minimal ages, but in absence of further evidence such upper bounds are technically advantageous for preventing the root of the tree to assume unreasonably old ages.

The ITS sequences generated here for 14 *Hoffmannseggella *species were completely re-aligned with those of van den Berg *et al. *[30], along with outgroup sequences from the 'Higher Epidendroids' downloaded from *GenBank*. The alignment was performed using MAFFT version 5.64 [47]. The age for the root of the tree was set to a normal prior distribution with mean 39 Ma and standard deviation of 5.5 (giving a 95% CI ranging from *c*. 31 - 49 Ma) corresponding to the resulting age estimate for the 'Higher Epidendroids' in the second analysis of the Orchidaceae matrix (see under Results).

The Yule process was chosen as speciation process for all three data sets. The Akaike Information Criterion in MrModelTest v2.3 [53] was used to choose the best-fitting evolutionary model for each sequence region (GTR+Γ+I for both partitions in the Orchidaceae data set and GTR+Γ for ITS in the *Hoffmannseggella *data set). Five separate runs were performed in BEAST with 20 million generations each. Log files were analysed with Tracer v1.5 [45], to assess convergence and confirm that the combined effective sample sizes for all parameters were larger than 200, in order to ensure that the MCMC chain had run long enough to get a valid estimate of the parameters [54]. All resulting trees were then combined with LogCombiner v1.5.3, with a burn-in of 25%. A maximum credibility tree was then produced using TreeAnnotator v1.5.3 [21].

## Abbreviations

BEAST: Bayesian evolutionary analysis by sampling trees; CI: confidence interval; HPD: highest posterior density; Ma: million years (from mega-annum); MRCA: most recent common ancestor; PL: Penalized Likelihood.

## Authors' contributions

ALSG and AA designed the study; ALSG and CFV conducted fieldwork; ALSG generated molecular data and conduced data analysis; ALSG and AA interpreted the results and wrote the paper. All authors read and approved the final manuscript.
